# Using computational models to relate structural and functional brain connectivity

**DOI:** 10.1111/j.1460-9568.2012.08081.x

**Published:** 2012-07

**Authors:** Jaroslav Hlinka, Stephen Coombes

**Affiliations:** 1 Institute of Computer Science, Academy of Sciences of the Czech RepublicPod Vodarenskou vezi 271/2, 182 07 Prague 8, Czech Republic; 2 School of Mathematical Sciences, University of NottinghamNottingham, UK

**Keywords:** brain disease, computational modelling, functional connectivity, graph theory, structural connectivity

## Abstract

Modern imaging methods allow a non-invasive assessment of both structural and functional brain connectivity. This has lead to the identification of disease-related alterations affecting functional connectivity. The mechanism of how such alterations in functional connectivity arise in a structured network of interacting neural populations is as yet poorly understood. Here we use a modeling approach to explore the way in which this can arise and to highlight the important role that local population dynamics can have in shaping emergent spatial functional connectivity patterns. The local dynamics for a neural population is taken to be of the Wilson–Cowan type, whilst the structural connectivity patterns used, describing long-range anatomical connections, cover both realistic scenarios (from the CoComac database) and idealized ones that allow for more detailed theoretical study. We have calculated graph–theoretic measures of functional network topology from numerical simulations of model networks. The effect of the form of local dynamics on the observed network state is quantified by examining the correlation between structural and functional connectivity. We document a profound and systematic dependence of the simulated functional connectivity patterns on the parameters controlling the dynamics. Importantly, we show that a weakly coupled oscillator theory explaining these correlations and their variation across parameter space can be developed. This theoretical development provides a novel way to characterize the mechanisms for the breakdown of functional connectivity in diseases through changes in local dynamics.

## Introduction

Modern brain imaging methods allow the quantitative study of both local activity dynamics and the interdependency between activities in anatomically distant areas. The latter, known as functional connectivity (FC) analysis, is of growing interest in the clinical and experimental neuroscience community.

Functional connectivity refers to the temporal synchronization of neural activity in spatially remote areas. It is widely believed to be significant for the integrative processes in brain function. FC is typically assessed using brain activity data acquired during a relaxed resting condition, although it can also be assessed from measurements taken during a particular task. The resting condition poses minimal demands on experimental preparation and the measured subject whilst still providing reliable information about a range of brain networks (Shehzad *et al.*, 2009; Smith *et al.*, 2009).

In practice, it is evaluated using a range of statistical techniques fitted to the particular measurement modality, including high temporal resolution methods such as electroencephalography and magnetoencephalography as well as high spatial resolution methods such as functional magnetic resonance imaging. For the latter, simple linear dependence measures (linear correlation) are suitable (Hlinka *et al.*, 2011a) while for the former, more time-resolved, data modality, elaborate methods of synchronization assessment such as mean phase coherence (quantifying the phase synchronization) are commonly used.

As malfunction of integration of neural information, further affecting cognitive and emotional processing, is believed to be central to many psychiatric and neurological diseases, a range of studies has investigated the differences in FC in patients with specific disease compared to healthy subjects. Although the description of such differences is still far from complete, many specific results for particular diseases have already been reported. We refer the reader to the Discussion section and recent reviews for more details (Bassett & Bullmore, 2009; Sporns, 2011).

While FC analysis as a data processing method seems to be effective in the detection of consistently synchronized networks of brain areas, there is an ongoing discussion regarding the origin of the specific observed patterns. The very structure of the anatomical connections between remote brain areas, termed structural connectivity (SC), is the most widely discussed potential contributor to the observed spatial pattern of FC. However, the extent to which it determines the FC is not known (Honey *et al.*, 2010), with reports of various degrees of predictability (see Ghosh *et al.*, 2008; Daffertshofer & van Wijk, 2011; or Honey *et al.*, 2010 and references therein).

In view of the situation described above, we contribute in this paper to the discussion of the relation between SC and FC by describing the role of mutual synchronizability of brain subunits modelled as neural oscillators. We have illustrated this general argument by systematically studying changes under parametric variation in a network model based on coupled neural populations. We have further assessed the associated changes in the FC topology, relating this to the hypothesis of increased randomization of brain networks in disease. Finally, we have related the results to theory-based predictions of changes in global network synchrony.

## Materials and methods

### Model description

To investigate the relationship between SC and FC in brain networks, we constructed a phenomenological model using standard components. In particular, we considered a network of interacting neural populations, coupled by a specific matrix of connections.

For simplicity, we assumed a parcellation of the cerebral cortex into functional areas, such that each area corresponds to a functional unit that can be represented by a single instance of a localized neural population model. We also assumed that the approximate structure of neural connections between the functional areas was available in the form of a connectivity matrix.

Each cortical area in the model is represented by a Wilson–Cowan (Wilson & Cowan, 1972) node – a model of two interacting populations of neurons. This represents a simple (but historically important and well recognised) example of an oscillatory neural population model, convenient here for ease of introduction, analysis and simulation. In principle for detailed simulations it could be replaced with other choices of neural mass models such as developed by Jansen & Rit (1995), Marten *et al.* (2009) and Liley *et al.* (2010), which are more amenable to accurately describing real EEG and fMRI time-series data. Denoting the activity of the two Wilson–Cowan local populations by *u* and *v* the network equations are written as:


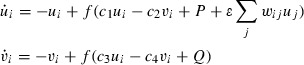
(1)

where *f*(*x*) = 1/(1 + exp (−*x*)) and represents a population firing rate function. Here *w*_*ij*_ > 0 with *w*_*ii*_ = 0 and reflects the anatomical (structural) activity pattern in a system of *N* nodes [each described by the pair (*u*_*i*_, *v*_*i*_)]. This is defined by the matrix *b*_*ij*_ with entries 0 or 1 where we set 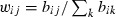 (so that the input to each node is normalised). The constants *c*_1_, …, *c*_4_ denote the strength of interaction between sub-populations within a node. For the current analysis we chose *c*_1_ = *c*_2_ = *c*_3_ = 10 and *c*_4_ = −2 as in Hoppensteadt & Izhikevich (1997). For ɛ = 0 the network dynamics decouples into a set of identical nodes, each of which may oscillate or be at rest depending on initial data and the values of the system parameters.

### Bifurcation analysis

**FIG. 1:**
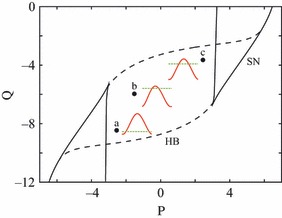
Bifurcation diagram for an isolated Wilson–Cowan node in the (*P*, *Q*) plane. Here HB denotes Hopf bifurcation and SN a saddle node of fixed-points bifurcation. Here *c*_1_ = *c*_2_ = *c*_3_ = 10 and *c*_4_ = −2. Also shown are plots of *H*(*θ*) (solid red line) at three different points in the (*P*, *Q*) plane. At points a ((*P*, *Q*) = (−2.5, −8.5)) and c ((*P*, *Q*) = (2.5, −3.5)) we find *H*’(*θ*) < 0 and at point b ((*P*, *Q*) = (−1.5, −6)) *H*’(*θ*) > 0. The green dashed line is the *θ* axis (*H* = 0). A breakdown of FC (loss of synchrony) is predicted at points a and c (for weak coupling between nodes).

A linear stability analysis of the node dynamics shows that oscillations may arise via a Hopf bifurcation. The locus of these Hopf bifurcations (HB) in the (*P*, *Q*) parameter plane is shown in Fig. 1, as well as the locus of saddle-node (SN) bifurcations of fixed-points (as the system may have either one or three rest states). The diagram shows only the main skeleton of the bifurcation structure of the system (though see Hoppensteadt & Izhikevich, 1997, for more detail).

### Structural connectivity

**FIG. 2:**
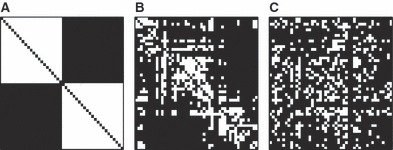
SC matrices for (A) modular connectivity, (B) brain anatomical connectivity (CoComac database; Kotter, 2004) and (C) random connectivity with the conserved degree sequence of (b). A nonzero entry at the position with coordinates *i*, *j* denotes the existence of anatomical link from the *j*-th to the *i*-th network node.

For exploration of the relation between SC and FC, we considered three types of connectivity matrices. The first is modular connectivity, where the network consists of several modules that are fully connected inside and have no connections between them. The second is an example of a quasi-realistic scenario, where we chose the parcellation of the cerebral cortex and the SC matrix in agreement with Honey *et al.* (2007) as 47 areas of macaque cortex together with an anatomical connectivity matrix collated in the CoComac database (Kotter, 2004). The third is a random binary matrix produced by the Maslov–Sneppen algorithm (Maslov & Sneppen, 2002), preserving the number of connections of each node (degree sequence) and therefore also the overall network density of the macaque matrix. The structural matrices are shown in Fig. 2.

### Simulations of the model

The dynamics of the model were simulated using in-house Matlab scripts. To facilitate the detection of stable solutions, a small amount of additive white Gaussian noise with variance *σ* = 0.01 was added independently to the *u* variable of each node and the system integrated using the Euler–Murayama method, with a time step d*t* = 0.1. Parameters *P* and *Q* were varied in the intervals (−6, 6) and (−12, 0) respectively with increments of 0.25. The coupling strength was fixed to 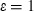.

**FIG. 3:**
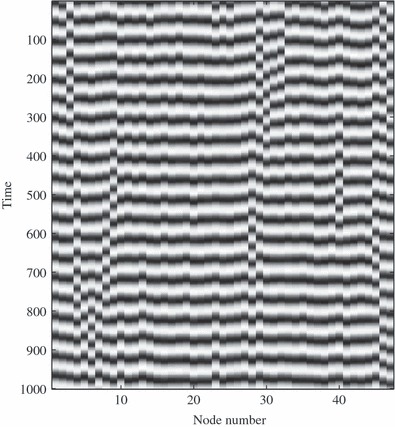
Example segment of time series of the model for a network of 47 population nodes connected by the CoComac network of connections; the u-variable is shown for each node.

For each parameter setting, a run of the model of length *T* = 10 000 was simulated, with random initial conditions for *u* and *v* variables of all nodes chosen uniformly from the interval (0,1). For FC analysis, the first 1000 steps were discarded to allow for initial transients. An example of the system behaviour is shown in Fig. 3. All the local populations show oscillations, with a typical period of approximately 50 time units.

### Measurement of FC

For assessment of FC we employed two commonly used measures. As a primary measure, the simple linear (Pearson's) coefficient of correlation of the time series was used. As the model supports nonlinear oscillations, we also computed the mean phase coherence (Mormann *et al.*, 2000). The computation of mean phase coherence requires first determining the instantaneous phase of each signal at every time point, which was carried out by applying a Hilbert transform and reading out the angle of the complex output. The phase coherence of the two signals is then quantified as the temporal stability of the phase difference *ϕ*_diff_(*t*) = *ϕ*_1_(*t*) - *ϕ*_2_(*t*). The mean phase coherence thus is defined as:


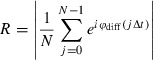
(2)

and we refer the reader to (Mormann *et al.*, 2000) and the references therein for details.

### Graph–theoretical measures

For the purposes of studying functional networks, the graph–theoretic approach is commonly used (Bullmore & Sporns, 2009). Here we used standard methods to compute some of the most commonly used graph–theoretic network characteristics (:) average path length, clustering coefficient and small-world index. We include only a brief description; see Bullmore & Sporns (2009) for details.

First, the FC matrix was binarized by choosing a threshold and keeping only links that held above-threshold connectivity values. For our simulations, we chose the value of the threshold such that the density of the resulting graph was equal to that of the underlying SC.

One can compute a distance matrix ***D***_*ij*_ holding for each pair of nodes the length of the shortest connecting path (minimal number of links necessary to go through to get from i to j). By averaging this over all node pairs, one obtains the average path length *L*, one of the key graph–theoretical measures.

For each node one can also compute its local clustering coefficient *c*, defined as the ratio of the existent links between its neighbours to the total number of such possible links. By averaging this coefficient over all nodes, one obtains the (global) clustering coefficient *C*.

To facilitate comparability, these characteristics are often related to the expected values for a corresponding random graph (commonly the Erdos–Renyi random graph, conserving the number of links, is used.). Thus we obtained relative coefficients 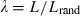 and *γ* = *C*/*C*_rand_.

A widely discussed network property is so called small-worldness; see Watts & Strogatz (1998) for an original discussion of the concept. A key property of a ‘small-world’ network is that it has similar average path length, but increased clustering coefficient compared to a corresponding random graph. These properties are summarized by the small-world index 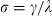 suggested in Humphries & Gurney (2008).

### SC–FC agreement

The level to which SC overlaps with the FC can be conveniently captured by the Jaccard similarity coefficient of the non-diagonal entries of the binary SC and FC matrices. This is the relative number of links that are shared by the SC and FC matrices with respect to the total number of links that appear in at least one of the matrices. Such a ratio is a natural measure of connectivity matrix overlap, ranging from 0 for matrices with no common links to 1 for identical matrices.

### Computation of stability

To provide a theoretical background and interpretation of numerical results, we consider the following theoretical arguments.

Synchronization phenomena in neuroscience have been extensively studied from a theoretical perspective using weakly coupled oscillator theory (Hoppensteadt & Izhikevich, 1997). This theory allows us to predict the stability of specific dynamic oscillatory network solutions. In the following we outline the method, and use it to derive a heuristic model for the prediction of parameter sets that will yield a high degree of correlation between SC and FC.

### Transformation of the model

We may rewrite the model given by Eqn (1) in the matrix form


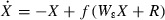
(3)

with *X* = (*u*_1_, …, *u*_*N*_, *v*_1_, …, *v*_*N*_), *R* = (*P*, …, *P*, *Q*, …, *Q*) and


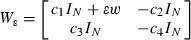
(4)

where *w* has components *w*_*ij*_ and *I*_*N*_ is the *N* × *N* identity matrix so that *W* = 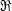
^2*N*×2*N*^. Using the linear transformation *Y* = *WX* + *R* we obtain a similar model, though with the term in *ɛ* appearing additively:


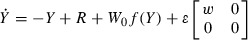
(5)

This is in a more convenient form for applying standard phase reduction techniques.

### Phase reduction

Writing *Y* in component form as *Y* = (*x*_1_, …, *x*_*N*_, *y*_1_, …, *y*_*N*_) means that Eqn. 5 takes the form


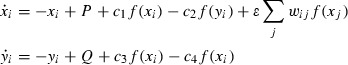
(6)

For ɛ = 0 we look for oscillatory solutions with a common trajectory such that (*x*_*i*_(*t*), *y*_*i*_(*t*)) = (*x*(*t* + *θ*_*i*_*T*), *y*(*t* + *θ*_*i*_*T*)), for some arbitrary phase-shifts *θ*_*i*_ 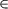 [0, 1), where (*x*(*t*), *y*(*t*)) is a *T*-periodic solution of


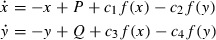
(7)

For weak coupling (small ɛ) we may then invoke the theory of weakly coupled oscillators to obtain a description of the network dynamics in terms of a set of phase variables that evolve according to


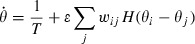
(8)

for *i* = 1, …, *N*. Here the phase interaction function (which is 1-periodic) is given by a temporal average of the product of the phase response vector of oscillator *i* and the interaction from oscillator *j*. The former can be found once the periodic orbit has been determined (by solving the so-called adjoint equation) and the latter merely requires writing *f*(*x*_*j*_) in the form *f*(*x*(*t* + *θ*_*j*_*T*)). The numerical machinery for constructing the phase interaction function is conveniently implemented in XPPAUT (Ermentrout, 2002). Note that this approach differs significantly from that in Daffertshofer & van Wijk (2011), which constructs a phase-oscillator network by linearizing around an unstable fixed point. Instead we have used the notion of phase response, which is the appropriate technique for describing systems with large amplitude oscillations such as those seen in Wilson–Cowan networks.

From the set of ordinary differential Eqn (8) it is particularly easy to determine the stability of the synchronous state (*θ*_*i*_ = *θ* for all *i*), which is key in determining FC. We introduce the *N* × *N* matrix 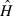 with components:


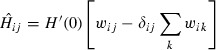
(9)

where *H*′(*θ*) = *dH*(*θ*)/*dθ*. The stability of a general phase-locked solution of the system of oscillators depends on the eigenspectra of the Jacobian 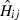 of the linearized perturbation equation. In particular, while one eigenvalue is always zero, the solution is stable if all the remaining eigenvalues have negative real parts. For a synchronous solution, the phase differences are all zero. A sufficient condition for the instability of the synchronous state is that Tr 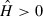 (as this would require at least one eigenvalue to have positive real part). Given that *w*_*ij*_ > 0 and *w*_*ii*_ = 0 we would therefore expect to see a breakdown in global synchrony if *H*′(0) < 0. Similarly, for a globally coupled network (∀*i* ≠ *j*; *w*_*ij*_ = *c*), the matrix has a very simple form and the non-zero eigenvalues are easily shown to be −1 (with *N*−1 degeneracy). Therefore, the synchronous solution is stable if *H*′(0) > 0 and unstable if *H*′(0) < 0.

This provides us with conditions for global synchrony, that is, synchrony of all neural oscillators. However, we are mostly interested in complex patterns of partial synchrony. Computation of conditions for these is impractical, as these differ in principle for each particular synchrony pattern and each underlying SC. Nevertheless, the above analysis can provide a heuristic condition regarding the stability of synchronous patterns in agreement with the underlying SC.

In particular, the same stability condition *H*′(0) > 0 as for globally coupled networks also trivially holds for an isolated sub-network of two coupled oscillators. Thus wherever *H*′(0) > 0 holds in parameter space, the nodes connected by a structural link will tend to synchronize. On the other hand, we expect to see a disagreement between SC and FC if *H*′(0) < 0.

Some examples of *H* (obtained using XPPAUT) are shown in Fig. 1. At the points labelled a and c we predict a breakdown of FC [as *H*′(0) < 0], which is consistent with our direct numerical simulations of the full model. For more extensive computation of *H*′(0) as a function of parameters *P* and *Q*, an in-house interface to the Matlab-based continuation package MATCONT (http://www.matcont.ugent.be/) was used.

### Heuristic prediction of SC–FC agreement

For networks with complex SCs, total agreement of SC and FC may be impossible. Moreover, many candidate phase-locked solutions might be available for testing, rendering detailed treatment cumbersome. Nevertheless, we propose that a general tendency towards agreement between SC and FC might be determined from the pair-wise phase interaction function. In particular, we conjecture that when the phase interaction function is such that the bi-synchronized solution is stable, this would promote synchrony within those pairs of nodes in the networks that are coupled, further leading to increased agreement between SC and FC.

## Results and statistical analyses

### SC–FC agreement

**FIG. 4:**
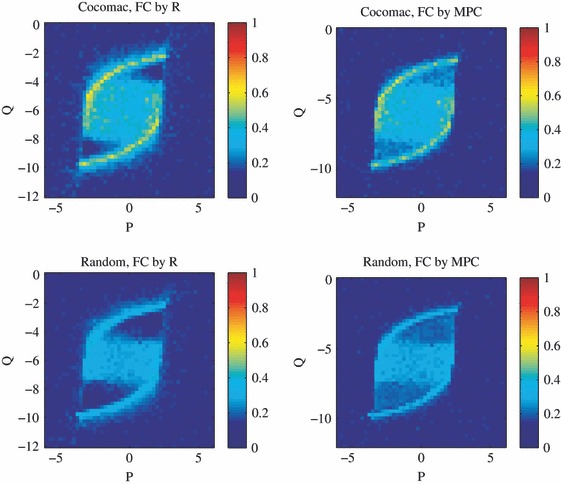
Agreement between the SC and FC as measured by the Jaccard similarity coefficient of the connectivity matrices. (Left) Measuring FC by correlation; (right) measuring FC by mean phase coherence; (top) CoComac SC; (bottom) random SC.

For all three types of SC, the variation of the SC–FC agreement within the (*P*, *Q*) parameter plane in the numerical simulations showed somewhat noisy but clearly non-random structure, which was to a large degree stable across the SC types and FC measures. See Fig. 4 for values of the agreement.

### Graph–theoretic properties of FC

**FIG. 5:**
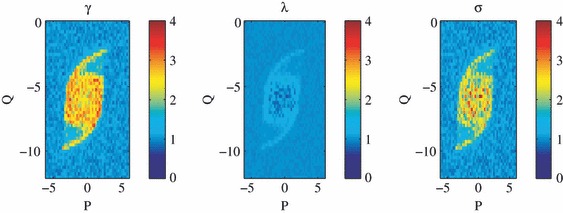
Graph–theoretical properties of FC measured by mean phase coherence as function of parameters *P* and *Q* of the Wilson–Cowan model.

The network-level effects of variation of the FC within the (*P*, *Q*) plane were assessed using a selection of commonly used graph–theoretic measures. Again, there was a clear structure of the dependence on (*P*, *Q*) parameters, affecting strongly the clustering coefficient and also the characteristic path length, leading to changes of the small-world index *σ*; see Fig. 5. The (*P*, *Q*)-dependence pattern was related to the one observed for the SC–FC agreement. In particular, the areas of increased clustering and small-world index (with respect to random network) generally overlapped with the areas of increased SC–FC agreement.

### Stability of synchronized solution

**FIG. 6:**
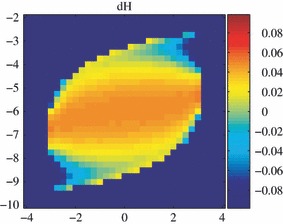
Stability of synchronous solution for weakly coupled network of Wilson–Cowan oscillators with global coupling. *H*′(0) is shown; note that its positive value corresponds to stability.

Numerical computation of the pair-wise phase interaction function outlined in Materials and Methods allows the determination of the stability of the fully synchronized (in-phase) solution. In particular, the positive sign of *H*′(0) corresponds to stability while a negative sign of *H*′(0) to instability of synchrony. The values of *H*′(0) as function of *P* and *Q* are plotted in Fig. 6, suggesting an overall large central area of synchrony instability with two roughly triangular areas of instability, at the lower left and upper right corner of the limit cycle region delineated by the bifurcation lines.

**FIG. 7:**
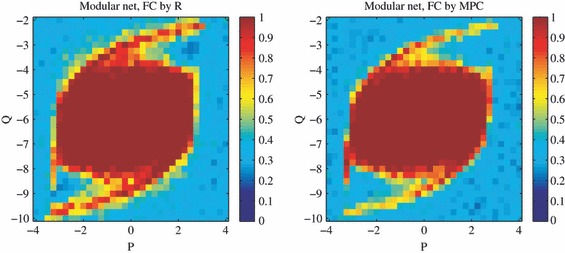
Agreement of FC and SC for a purely modular network as a function of model parameters *P* and *Q*. (Left) Measuring FC by correlation; (right) measuring FC by mean phase coherence. Note the good agreement with Fig. 6 (stability of synchronous solution), which can be directly proven for weak coupling.

For a network consisting of two independent and internally fully coupled modules, synchrony among all nodes within each module is from theory given by the sign of *H*′(0), while the synchrony between them is predicted as random and asymptotically zero as they are completely independent. This theoretical argument predicts perfect agreement of the FC matrix with the underlying structure for positive *H*′(0) and zero agreement for negative *H*′(0). This is confirmed by simulations; see Fig. 7.

Note that this also suggests that the strength of coupling ɛ = 1 is sufficiently small for the predictions of the weak coupling theory to approximately hold.

## Discussion

The current study has documented how FC within a network of neural populations crucially depends on the parameters of the populations. Moreover, the drastic consequences of this dependence for the agreement between SC and FC, as well as for the global functional network properties, has been investigated. Importantly, a link to the theory of weakly coupled oscillators has been established. In particular, in the parameter regions where the synchronous solution is predicted to be stable, the FC resembles the underlying SC. On the other hand, in areas of instability of synchronous solutions (for pair-wise or fully coupled networks) the network shows a richer pattern of activity, generally independent of the underlying structural connections. While several specific choices had to be made for the purpose of this study, we expect that the main results hold under more general conditions.

There is an increasing interest in analyzing brain FC alterations using graph–theoretical approaches. For example, such techniques have been applied to electrophysiological data for a variety of diseases including Alzheimer's dementia (Stam *et al.*, 2007), major depression (Leistedt *et al.*, 2009) and schizophrenia (Rubinov *et al.*, 2009). We refer the reader to recent reviews for more details (Bassett & Bullmore, 2009; Sporns, 2011). In particular the latter review highlights the notion of randomization (loss of specific complex properties of brain connectivity) as an important potential common mechanism for FC disruption in brain disease. As various diseases have been shown to have such randomizing effects on brain FC, the instability arising from variation in population properties may have direct relevance for understanding the way diseases affect brain activity dynamics, being a candidate mechanism for FC disruption in the absence of (or as a precursor to) notable changes in the anatomical connectivity or local dynamics.

### Model parameter settings

Within our high-level model, the values of some parameters could not be directly informed by biology. The strength of the coupling ɛ was chosen for convenience (and set to unity). For weaker coupling, longer time series would have to be simulated to achieve robust statistical sampling of the process, which would pose an impractical demand for both memory and computational time especially in combination with the requirement to cover a two-parameter plane. Importantly, even though the coupling strength was not ‘negligible’, the weak coupling theory stability prediction still proved relevant for understanding the FC topology. A small amount of noise was also implemented in the model. We have not investigated the parametric variation of the results with noise levels so only note that, while we have obtained similar results for decreased or moderately increased noise levels, under very strong noise, synchronisation is, as expected, less robust.

The role of node heterogeneity in the formation of FC has recently been studied by Daffertshofer & van Wijk (2011). We have observed very similar results for both a network with homogenous nodes and a network where a random fluctuation in the *P* parameter drawn from interval (0, 0.01) was added to each node. This confirms that the effect does not require strictly homogeneous node properties. As the node heterogeneity introduces several more degrees of freedom, which would require additional space to explore and describe, the role of the heterogeneities will be reported upon elsewhere.

### Model choice

In the Wilson–Cowan model considered, the parameters *P* and *Q* were conveniently chosen for exploration of the dependence of FC on parameter variation. In the original formulation, these correspond to the background level of non-specific input to the two node subpopulations. In this context our findings could be interpreted as the potential effect of global increase or decrease in excitatory or inhibitory transmission, leading to deviation from the optimal modus of function and breakdown or randomization of FC pattern. More generally, *P* and *Q* can be interpreted as general modulators of the local input response.

### FC assessment

The choice of methods for FC assessment is still a matter of intense research and debate even within the experimental community. The mean phase coherence used in this paper is among those most widely used for quantification of dependence of electrophysiological signals. On the other hand, while many nonlinear methods have been recently tested in this respect, it has been confirmed that linear correlation is generally sufficient for the assessment of FC for functional magnetic resonance imaging data (Hlinka *et al.*, 2011a). Our model output is generally closer (though not equivalent) to a local field potential and therefore means phase coherence is the method of first choice. Nevertheless, we also include the results for the linear correlation measure as it is widely known and also commonly used in studies of interactions within complex dynamic systems. In general, the results using both measures have shown marked similarities, although they also offer to some extent complementary information due to their differential sensitivity with respect to out-of phase synchrony. In particular, linear correlation can show negative values for prevalently anti-phase synchronized oscillations while mean phase coherence can show positive ones, reflecting the strength of synchronization. The differences between these measures constitute a still widely neglected but important subject for further study.

### Graph–theoretic network characteristics

In the present study, the overall effect of changing system parameters on the FC pattern has been studied on several levels. Apart from the consequences for the structure–function relationship, the global topology alterations have been probed using a selection of widely used graph–theoretic measures. There was a pronounced variability particularly in the clustering coefficient and small-world index with respect to the degree-matched random graph, suggesting a potential mechanism for the breakdown of FC towards increased randomness in specific areas.

The binarization threshold for the FC was conveniently chosen to preserve the density of the structural matrix. Although a change in the threshold would lead to quantitative alterations in the graph–theoretical measures, the specific threshold choice (within reasonable limits) does not strongly affect the general qualitative results that we obtained.

Of note, although graph–theoretical approaches are commonly applied for the characterisation of both SC and FC and are believed to provide important qualitative and quantitative information about the topology of the corresponding graphs, the interpretation of the path-based measures in FC matrices is not as straightforward as with SC due to the abstract nature of these paths. The problem is discussed in more detail in Rubinov & Sporns (2010).

It has recently also been shown that a FC approach using common measures introduces some bias into the graph topology, namely a spurious tendency towards increased clustering and small-worldness (Hlinka *et al.*, 2011b). However, this is not crucial in the current context as long as it is the parameter-dependent relative variation rather than the exact quantitative values of the graph measures that is interpreted.

The specific interpretation of graph–theoretic measures in the context of brain function is an ongoing topic of research outside the scope of the current paper; see e.g. Bullmore & Sporns (2009) and Rubinov & Sporns (2010) for reviews.

### Relating SC and FC

We have observed that the agreement between SC and FC was crucially modulated by the parameter regime. In particular, in agreement with our intuitive theoretical argument, high values of agreement were observed in the analytically derived parameter area corresponding to pairwise synchronizability of local dynamics, while low values were observed in areas that correspond to instability of pairwise synchrony (compare Fig. 6 with Fig. 4).

For purely modular networks (Fig. 7), this agreement was perfect up to some noise due to finite-size simulation effects. For a random network, the patterns were qualitatively similar as for a more realistic brain network, although the prediction was weaker. This is probably related to the tendency towards modular architecture in real brain networks; see also Honey *et al.* (2007). The specific role of modularity in the formation of FC is a complex topic requiring further research. The robustness of the brain network results was confirmed with additional simulations using an alternative SC matrix (cat cortex; see Scannell *et al.*, 1999); see Fig. S1.

Note that the similarity of matrices can be quantified by a range of methods. The Jaccard similarity coefficient is preferable for its conceptual simplicity (effectively being a relative number of shared links). Although only results using the Jaccard similarity coefficient are presented in this paper, other measures such as Pearson correlation coefficient were tested in an exploratory fashion and offered qualitatively similar results.

In general, experimental studies support the idea that structural connections, when present, are substantially predictive of the presence or strength of functional connections, although this relation may be only moderate in available data. In computational models, even stronger agreement of SC and FC has been reported in some cases; see e.g. Ghosh *et al.* (2008), while the potential for their decoupling has been discussed elsewhere (Daffertshofer & van Wijk, 2011).

Although the investigation here was not aimed at fitting a particular experimental dataset, we were able to confirm that the values of the structure–function agreement in the synchronization regime were of similar order as those generally reported in the experimental literature, suggesting substantial, but far from perfect, agreement.

Experimental establishment of the relation of structure and dynamics (or function), though progressing, is still complicated by methodological difficulties with *in vivo* measurements of SC (Hagmann *et al.*, 2008). On the other hand, modeling studies are relatively scarce, include specific and widely differing assumptions and therefore offer only partial answers. The isolated reports differ in whether they predict high similarity between SC and FC as well as in the expected reliability of this prediction; see e.g. Daffertshofer & van Wijk (2011), Honey *et al.* (2010) and Ghosh *et al.* (2008).

Thus, understanding the potential and observed changes in FC in disease would benefit from embedding previous studies into a broader framework, the aim being an improved theoretical understanding of the principles of the emergence of FC patterns from the dynamic activity of local nodes coupled through long-range anatomical connections.

In this context, it seems reasonable to assume that the extent of the relationship might in fact strongly depend on some key system parameters. In this paper we document such dependence using a basic computational neuroscience model and provide a tentative explanation of a major source of this variability through the stability analysis of synchronized behaviour.

The argument presented is applicable to a wide class of oscillator-based neural models. Importantly, the desynchronization mechanism of FC disruption we have described, through its decoupling from the SC substrate, may play a role in the topological changes observed in brain FC in disease. In particular, the topology of FC was shown to potentially drastically change without a notable change of the underlying structural substrate or local dynamic behaviour.

For simplicity we have not included axonal transmission delays in the model. However, these can potentially play a significant role in shaping FC, particularly for resting-state networks. For a review we refer the reader to Deco *et al.* (2011). Moreover, the inclusion of delays in neural mass models is known to allow for a richer repertoire of response, including chaotic behaviour (Coombes & Laing, 2009). In the phase-reduced description the presence of a delay would manifest itself as a phase shift. For small enough delays this can be described by a phase shift in the phase interaction function, as in Daffertshofer & van Wijk (2011). Interestingly, time delays can cause the stability of the synchronous state to switch periodically as they are increased (Campbell & Kobelevskiy, 2012).

The Wilson–Cowan model was chosen as a widely known example of a neural population model with exemplary oscillatory dynamics, also suitable for its simplicity. Of course, more sophisticated and biologically realistic models would have to be studied to provide more specific, potentially quantitative, predictions regarding the effects of variation of particular physiological variables due to brain disease or other factors modulating brain function. Related to this, it would be beneficial to complement this work in future with more accurate forward models that connect the local neural activity to the variables observed in experimental brain measurements using diverse brain imaging methods (Bojak *et al.*, 2010). In such detailed modelling, realistic levels of noise and signal conduction delays should be implemented; they have already been shown to contribute to the reproduction of realistic FC patterns in specific models (Ghosh *et al*., 2008).

The described phenomena leave many open questions. For instance, while we have shown that the predictive power of pair-wise synchrony stability for the structure–function agreement is strong across widely varying topologies, it may be parametrically modulated by the network topology or other parameters of the dynamic model. For instance, this prediction should intuitively be very precise for sparse networks formed by many isolated node doublets, while for denser and more complex connectivity matrices it should provide rather an approximate heuristic estimate (and we should instead analyze the full Jacobian matrix of the system); this dependence may be of interest for the investigation of real data.

## Conclusion

In conclusion, the current paper contributes to the study of large-scale patterns of brain activity dynamics and its alterations in disease by documenting the profound effects of subtle changes of parameters within local nodes on the FC topology. For a specific model, we have identified regions of breakdown of the FC pattern characterized by increased randomization and decreased resemblance to the underlying structural coupling pattern. This was further related to theoretical predictions by correspondence to the parameter-regions of instability of pair-wise synchronous solutions. Further work is ongoing that will formulate a more rigorous theoretical framework for explaining FC patterns and their alterations in brain disease.

## Abbreviations

FC: functional connectivity
SC: structural connectivity

## Supporting Information

Fig. S1. Agreement between the structural and simulated functional connectivity as measured by the Jaccard similarity coefficient of the connectivity matrices. Left: quantifying functional connectivity by correlation; Right: quantifying functional connectivity by mean phase coherence; Top: cat cortex structural connectivity; Bottom: random structural connectivity. Visualization as in Figure 4 of the article that presents results for macaque structural connectivity matrix.

Please note: As a service to our authors and readers, this journal provides supporting information supplied by the authors. Such materials are peer-reviewed and may be re organized for online delivery, but are not copy-edited or typeset by Wiley-Blackwell. Technical support issues arising from supporting information (other than missing files) should be addressed to the authors.
